# Prevalence of Abortion and Contraceptive Practice among Women Seeking Repeat Induced Abortion in Western Nigeria

**DOI:** 10.1155/2015/486203

**Published:** 2015-05-19

**Authors:** Mustafa Adelaja Lamina

**Affiliations:** Maternal and Fetal Health Research Unit, Department of Obstetrics and Gynaecology, Olabisi Onabanjo University Teaching Hospital, PMB 2001, Sagamu, Nigeria

## Abstract

*Background*. Induced abortion contributes significantly to maternal mortality in developing countries yet women still seek repeat induced abortion in spite of availability of contraceptive services. The aim of this study is to determine the rate of abortion and contraceptive use among women seeking repeat induced abortion in Western Nigeria. *Method*. A prospective cross-sectional study utilizing self-administered questionnaires was administered to women seeking abortion in private hospitals/clinics in four geopolitical areas of Ogun State, Western Nigeria, from January 1 to December 31 2012. Data were analyzed using SPSS 17.0. *Results*. The age range for those seeking repeat induced abortion was 15 to 51 years while the median age was 25 years. Of 2934 women seeking an abortion, 23% reported having had one or more previous abortions. Of those who had had more than one abortion, the level of awareness of contraceptives was 91.7% while only 21.5% used a contraceptive at their first intercourse after the procedure; 78.5% of the pregnancies were associated with non-contraceptive use while 17.5% were associated with contraceptive failure. The major reason for non-contraceptive use was fear of side effects. *Conclusion*. The rate of women seeking repeat abortions is high in Nigeria. The rate of contraceptive use is low while contraceptive failure rate is high.

## 1. Introduction

Unintended or unplanned pregnancy poses a major economical, psychological, social, and/or religious challenge in women of reproductive age, especially in developing countries. It has been estimated that, of the 210 million pregnancies that occur annually worldwide, about 80 million (38%) are unplanned and 46 million (22%) end in abortion [1]. More than 200 million women in developing countries would like to delay their next pregnancy or even stop bearing children altogether [2], but many of them still rely on traditional and less effective methods of contraception or use no method at all.

In Nigeria, unintended intercourse is the primary cause of unwanted pregnancies, and many women with unwanted pregnancies decide to end them by abortion [3]. Since abortion is illegal in Nigeria (unless medically recommended to save a mother's life), many abortions are carried out clandestinely, and often in an unsafe environment [4]. Induced abortion is not only widespread in Nigeria but is also provided and practiced in a number of different settings, from traditional medical practitioners, herbalists, and private practicing clinicians to modern pharmacists [5]. The consequences of these clandestine abortions are grave and can be life-threatening, often leading to maternal death. Abortions account for 20%–40% of maternal deaths in Nigeria [4–6].

The leading contributory factor to unwanted pregnancy in Nigeria is low contraceptive usage [7–9]. The current prevalence rate for contraceptive use in Nigeria is approximately 11%–13% [10]. This rate is very low in spite of the high rate of sexual activity (the average age of sexual debut ranged between 12 and 20 years, with a mean age of 16 ± 1.2 years) and widespread awareness of the various contraceptive methods (ranging between 29% and 69% depending on the method) among Nigerian adolescents and youths [10, 11]. Several studies in Nigeria have shown that more than 60% of women with unplanned pregnancies were not using contraception. The consequence of low contraceptive use among Nigerian women leads to an estimated 1.5 million unplanned pregnancies every year, with half of these resulting in elective abortions [4, 12–14].

It has also been noted that some women use abortion as a means of child-spacing instead of using modern contraception [3]. Fear of future infertility was the overriding factor in adolescents' decision to rely on abortion rather than contraception [3]. Many perceived the adverse effect of modern contraceptives on fertility to be continuous and prolonged, while abortion was seen as an immediate solution to an unplanned pregnancy [3].

Hence, the aim of this study was to determine the rate of repeat induced abortions and the related contraceptive practices among women in private health care facilities in Western Nigeria.

## 2. Subjects and Methods

### 2.1. Study Setting

Nigeria has a population of about 170 million people, 70% of whom live in rural areas [9]. Ogun State as one of the 36 states in Nigeria is located in the western region of the country. Ogun State has a land area of 16,762 square kilometers and a population of a little over 1 million. It has 20 local government areas, six urban and 14 rural. The Institutional Ethics Review Committee of Olabisi Onabanjo University Teaching Hospital, Sagamu, Ogun State, Nigeria, gave the approval to conduct this study.

### 2.2. Study Design and Participants

The current study was a prospective hospital-based study. The sample was drawn from the list of private hospitals/clinics in Nigerian Medical Directory [15]. Thirty-two private health institutions were selected, 18 in urban and 14 in rural local government areas. These institutions were located in the most and least urbanized areas of local government in Ogun State. Every fourth hospital was selected in the urban local government areas while all the hospitals in the rural local government areas were chosen (because there were fewer hospitals in the rural areas than urban areas). The medical officers in these institutions who provided the abortion care were then approached and their cooperation solicited in view of the sensitive and legal nature of the procedure. They were assured of confidentiality and anonymity and the fact that the information obtained would be used for research purposes only.

A semistructured questionnaire was developed for the purpose of this study and was pretested among 25 abortion care seekers a month preceding the commencement of the study in 2011. After pretesting, the questionnaire was modified according to local traditions and cultural sensitivity. The questionnaire sought information on sociodemographic and educational characteristics of abortion care seekers; number of previous abortions; contraceptive practice; and reasons for not using contraception.

The medical officers administered a questionnaire to every abortion care seeker in the institutions from January to December 2012. Trained medical officer interviewers, who visited the hospitals regularly, assured that the forms were properly filled out. After this assurance, the forms were collected and double-checked by a senior doctor. The senior doctor took the filled forms to the principal investigator for storage. Each trained medical officer covered eight hospitals and there were two supervisors for all the twenty local government areas.

The participants were assured that information provided by them will be kept confidential and will not be used against them. They were also assured that their names will not appear on the questionnaires and their responses will not cause them any harm neither will it prevent them from receiving the best of care from the hospital.

### 2.3. Data Analysis

Data analysis was by both descriptive and inferential statistics at 95% confidence level using* SPSS software for Windows version 17.0*. Frequency tables were generated for relevant variables. Proportions were compared with the Pearson chi-square test. Relationships were expressed using odds ratio and confidence intervals.

## 3. Results

Of 2934 women seeking abortion within the one-year study period, 675 presented for repeat induced abortion, giving a rate of 23%.

The age range for those seeking repeated induced abortion was 15 to 51 years while the median age was 25 years. Majority of women (70.5%) seeking repeated induced abortion were aged less than 30 years while 43.1% were within 15 to 24 years of age (Table 1).

Of the 675 women seeking repeat induced abortion, 47.9% were single and 51.6% were married.  Table 2 shows a cross-tabulation of number of repeat abortions and marital status of women seeking repeat induced abortion. A woman presenting for second- or higher-order repeat induced abortion is statistically significantly more likely to be single than married (43% versus 22%, odds ratio = 2.67 {1.38 < OR < 5.20}, 95% confidence limits, *p*-value = 0.0015225).

All the women had some form of formal education, with 97.6% having a minimum of secondary education. The bulk of the women were students (33.2%) and traders (32%).

The number of previous abortions ranged from 1 to 6, with a mean of 1.44 and median and mode of 2 each, respectively. About one-third of the women (32.3%) seeking repeat induced abortion have had 2 or more previous induced abortions (Table 3).

Of those who had had one or more abortions, the level of awareness of contraceptives was 91.7% while only 21.5% used a contraceptive at their first intercourse after the procedure, and of the 145 who chose contraception, 87.6% used modern methods of contraception: emergency contraceptive, condom, oral contraceptive, and injectable contraceptive (Table 4). Out of 11.3% of the women that used emergency contraceptive (6.4%) and condom (4.9%), only 10.5% used them consistently and correctly; 78.5% of the pregnancies were associated with non-contraceptive use while 17.5% were associated with contraceptive failure (mainly due to incorrect/inconsistent use of condoms and emergency contraceptives).

The reasons for not using contraceptive among women seeking repeat induced abortion are as shown in Table 5. About 70% of the women did not use any contraception because of fear of side effects and lack of adequate information/misinformation about contraception. About 10% of the women did not use contraception as a result of objection from partner and family members.

**Figure 1:**
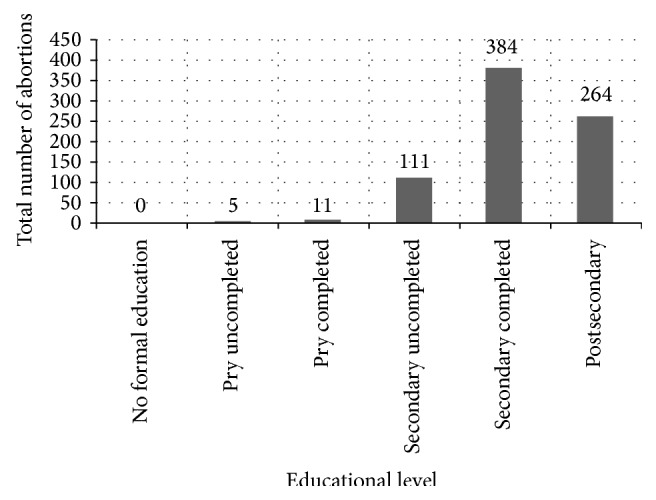
Educational level and total number of abortions of women with repeated induced abortion. Footnotes: pry uncompleted: primary school uncompleted; pry completed: primary school completed; secondary uncompleted: secondary school uncompleted; secondary completed: secondary school completed; postsecondary: postsecondary school.

**Figure 2:**
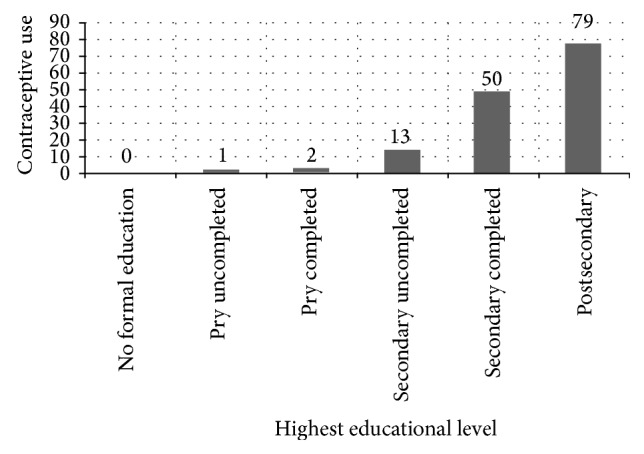
Educational level and contraceptive use of women with repeated induced abortion. Footnotes: pry uncompleted: primary school uncompleted; pry completed: primary school completed; secondary uncompleted: secondary school uncompleted; secondary completed: secondary school completed; postsecondary: postsecondary school.

Figures 1 and 2 show the relationship between education, number of abortions, and contraceptive usage of women presenting for repeat induced abortion.  Figure 1 shows the relationship between educational level and number of abortions in women presenting for repeat induced abortion. The number of repeat abortion increased with educational level.  Figure 2 shows the relationship between educational level and contraceptive usage of women presenting for repeat induced abortion. The contraceptive usage rate increased with increased educational level.

There is a direct relationship between the educational level of the women and the number of abortions and their contraceptive use. The number of abortions and contraceptive use increased with increase in educational level.

## 4. Discussion

Many Nigerian women of reproductive age experience an unwanted pregnancy and resort to abortion [3]. Unwanted pregnancy is the leading cause of unsafe abortion in Nigeria. Abortion law is restrictive in Nigeria. Therefore, induced abortions are carried out clandestinely, and sometimes under unsterile conditions and by unskilled personnel [10]. Abortions contribute to 20%–40% of all maternal deaths, constitute an economic drain on the Nigerian health system, and are expensive for women [16], especially for those who develop complications leading to pelvic inflammatory disease (PID), infertility, and/or ectopic gestation [16].

The rate of repeat induced abortion of 23% from this study is slightly lower than rates of 33% and 35% reported in China [17, 18]. However, having close to a quarter of women who had had one or more previous abortions presenting for repeat induced abortion is worrisome. This is a reflection of nonincorporation of postabortion contraceptive counseling in abortion care (as part of comprehensive abortion care), poor postabortion contraceptive counseling, or poor contraceptive uptake by the women presenting for repeat induced abortion [18].

More than two-fifths of the women presenting for repeat induced abortion were young women (age group within the 15–24 years) while adolescent constituted 11.6%. This rate in the adolescents is similar to 13.1% reported from a study in India [18] and lower than 23.7% in an earlier study from Nigeria [12]. This is attributable to nonconsensual sex, early initiation of sexual activity, high sexual activity, and low contraceptive use among the adolescents [12, 19, 20].

The observation that a significant proportion of women presenting for repeat induced abortion were youths (15–24 years) and the fact that a woman presenting for second- or higher-order repeat induced abortion is more likely to be single than married are worrisome. This is born out of the fact that the level of knowledge and awareness of contraception is high among the youths. But paradoxically, contraceptive utilization rate is low among the youths as reflected in this study and others [12, 17–20]. Ambivalence and discomfort among health providers in communicating with unmarried youths and providing contraceptives to them and their right to privacy and confidentiality are some of the factors militating against contraceptive uptake among the youths [10, 21].

However, this procedure is not exclusively an activity of unmarried/young women, as a significant number of older/married women in the reproductive age group also sought abortion. This is consistent with the findings of studies from South-Western Nigeria [5, 12]. This is a reflection of low contraceptive use among the older/married women who desired child-spacing and limited family size in view of present economic hardship in Nigeria [10, 12].

It has also been noted that some women use abortion as a means of child-spacing instead of using modern contraception [3]. Fear of future infertility was the overriding factor in adolescents' decision to rely on abortion rather than contraception [3]. Many perceived the adverse effect of modern contraceptives on fertility to be continuous and prolonged, while abortion was seen as an immediate solution to an unplanned pregnancy [3].

Majority of the respondents (97.7%) were aware of some methods of contraception. This compares well with 91.3% reported from South-Western Nigeria [5]. Despite this high level of awareness, only 21.5% of the women had tried contraception after the last abortion, showing a great gap between awareness and usage; the overall conclusion would be that knowledge does not necessarily translate into attitudinal change where contraception usage is concerned [22].

The reasons given by women presenting for repeat induced abortion for not using contraceptives in this study are similar to those reported in other studies [3, 7–9, 23]. These included fear of side effects, lack of adequate information/misinformation, objections from their partners, conflicts with their religious beliefs, objections from family members, not thinking about using contraceptives, not having sexual intercourse to have baby, and unplanned sexual intercourse. All the reasons depicted a basic problem, which is lack of proper information on contraception. Public enlightenment on contraception can start from antenatal and gynaecological clinics. This can be extended to other outpatient clinics which men are part of, so that the information on contraception will spread to as many people as possible. This will reduce the magnitude of misconception/misinformation on contraception to a minimum level and increase contraceptive use.

Despite the high association between education and usage rate of contraception, this same group also had a high association with abortion. This compares with similar findings from Lagos and other parts of the world, indicating that women who had used contraception are more likely to have had abortion than women who had not used contraception [5, 13, 24].

*Limitations*. The number of previous induced abortions could have been underestimated due to the fact that women are usually reluctant to own up to having had induced abortion. The effect of this factor has been minimized considerably by assuring and ensuring high degree of confidentiality and anonymity; and the fact that women easily volunteer previous history of induced abortion at antenatal clinics.

*Recommendations*. Lack or ineffective postabortion contraceptive counseling could contribute to high rate of repeat induced abortions. Therefore, there is a need to incorporate postabortion contraceptive counseling as part of comprehensive abortion care. In a study carried out in USA, it was reported that women having a second- or higher-order abortion were over twice as likely as women having a first abortion to indicate interest in long-acting reversible contraception [25]. Training of health care providers in abortion care is also a prerequisite to providing abortion care services of good quality. However, an advocacy for change in abortion policy in Nigeria (from being restrictive to liberal) is mandatory in order to provide a supportive environment for both health care providers and clients.

The low contraceptive utilization among the youths can be improved upon by providing adolescent-friendly sexual education and reproductive and contraceptive services to the unmarried youths. Moreover, evidence had shown that many unmarried young women presenting for repeat induced abortion were ready to receive postabortion contraception at abortion clinic [25].

There is also a need to translate high contraceptive awareness to an increased use in order to bridge the large gap of unmet need. There is a need to dismystify misinformation about contraception. A significant component of any family planning program for Nigeria would have to be concentrated on community health education to reduce misconceptions about the side effects of modern contraceptives, which is the most common reason for nonuse of modern contraceptives in Nigeria. Another component would be the involvement of men in family planning and use of the radio for information dissemination.

## 5. Conclusion

There is abundant information that contraceptive knowledge and awareness are high among the Nigerian population. This awareness has not been translated into contraceptive use, with the end-result being very low contraceptive prevalence in Nigeria. This low contraceptive prevalence correlates with high levels of unplanned pregnancies and abortions, leading to increases in the maternal mortality ratios, especially in the rural areas in Nigeria.

The medical technology is known, and the sociocultural, religious, family, misinformation, and male-dominant factors impeding contraceptive use in Nigeria societies have been identified. What is lacking is the generation of political priority for family planning and safe motherhood as well as the political will and commitment to make this change on a large scale. With the commitment of financial and human resources as well as assistance from international organizations, public-private sector collaboration, community-oriented knowledge, acceptability, and availability of a wide range of modern contraceptive choices, the contraceptive prevalence rate will increase and should contribute to the reduction of the prevalence of unwanted pregnancy and induced abortion and the worst maternal health crisis in Nigeria and sub-Saharan Africa. This will ultimately lead to substantial reduction in population growth and poverty reduction. At the existing rate of population increase, Nigeria will become a failed state because institutions cannot be built/maintained if the population keeps increasing with nearly 3% a year, which means a population doubling rate of a little more than 20 years [26].

## Figures and Tables

**Table 1:** The age distribution of women seeking first and repeat induced abortion.

Age group	First abortion seekers		Repeated abortion seekers	
(years)	Frequency	Percentage	Frequency	Percentage
15–19	142	6.3	78	11.6
20–24	703	31.1	213	31.5
25–29	626	27.7	185	27.4
30–34	391	17.3	101	15.0
35–39	296	13.1	61	9.0
40–49	99	4.4	35	5.2
50 and above	2	0.1	2	0.3

**Total**	**2259**	**100.0**	**675**	**100.0**

**Table 2:** Number of repeat abortions versus marital status of women seeking repeat induced abortion.

Number of previous abortion(s)	Marital status				Total
	Single	Married	Separated	Widowed	
1	183	270	1	3	457
2	103	60	0	0	163
3	27	13	0	0	40
4	6	4	0	0	10
5	4	0	0	0	4
6	0	1	0	0	1

**Total**	**323**	**348**	**1**	**3**	**675**

**Table 3:** Number of previous induced abortions.

Number of abortions	Frequency	Percentage	Cumulative percentage
1	457	67.7	67.7
2	163	24.2	91.9
3	40	5.9	97.8
4	10	1.5	99.3
5	4	0.6	99.9
6	1	0.1	100.0

**Total**	**675**	**100.0**	

**Table 4:** Contraceptive use among women seeking repeat induced abortion.

Method	Frequency	Percentage	Cumulative percentage
Emergency contraceptives	43	6.4	6.4
Condom	33	4.9	11.3
Oral contraceptive pills	31	4.6	15.9
Injectable contraceptives	18	2.6	18.5
Intrauterine contraceptive device	2	0.3	18.8
Rhythm method	5	0.7	19.5
Withdrawal method	2	0.3	19.8
Lactational amenorrhoea	1	0.2	20.0
Periodic abstinence	1	0.2	20.2
Traditional methods	9	1.3	21.5
No contraceptive method	530	78.5	100.0

**Total**	**675**	**100.0**	

**Table 5:** Reasons for non-contraceptive use among women presenting for repeat induced abortion.

Reason	Frequency	Percentage
Fear of side effects	291	54.9
Lack of adequate information/misinformation	79	14.9
Not thinking about using contraceptive	53	10.0
Objection from partner	31	5.9
Objection from family members	21	4.0
Conflicts with religious beliefs	5	0.9
Not having sexual intercourse to have a baby	36	6.8
Unplanned sexual intercourse	14	2.6

**Total**	**530**	**100.0**

## Appendix

### Prevalence of Abortion and Contraceptive Practice among Women Seeking Repeat Induced Abortion

*Semisructured Questionaire*

1. Age …
2. Marital status:
  - single
  - married
  - separated
  - divorced
  - widowed
3. Level of education:
  - none
  - primary
  - secondary
  - tertiary
4. Occupation …
5. Religion …
6. Parity …
7. Gestational age …
8. Age …
9. Marital status:
  - single
  - married
  - separated
  - divorced
  - widowed
10. Level of education:
  - none
  - primary
  - secondary
  - tertiary

(11) Occupation …
(12) Religion …
(13) Parity …
(14) Number of previous termination of pregnancy(ies) …
(15) Gestational age (weeks) at previous termination of pregnancy …
(16) Type of contraceptive used to prevent previous pregnancy:
  - male condom
  - oral pills
  - emergency contraception
  - rhythm method
  - withdrawal method
  - periodic abstinence
  - lactational amenorrhoea
  - intrauterine contraceptive device
  - injectable
  - traditional method
  - non
(17) Type of contraceptive used after previous termination of pregnancy:
  - male condom
  - oral pills
  - emergency contraception
  - rhythm method
  - withdrawal method
  - periodic abstinence
  - lactational amenorrhoea
  - intrauterine contraceptive device
  - injectable
  - traditional method
  - non
(18) Type of contraceptive ever used:
  - male condom
  - oral pills
  - emergency contraception
  - rhythm method
  - withdrawal method
  - periodic abstinence
  - lactational amenorrhoea
  - intrauterine contraceptive device
  - injectable
  - traditional method
  - non
(19) If no contraceptive was used, kindly state the reason …
